# Pyometra presenting in conjunction with bowel cancer in a post-menopausal women: a case report

**DOI:** 10.1186/1757-1626-1-24

**Published:** 2008-07-08

**Authors:** Hooman Soleymani majd, Sean Watermeyer, Lamiese Ismail

**Affiliations:** 1 Milton Keynes General Hospital, Department of Obstetrics & Gynaecology, Standing Way, Eaglestone, Milton Keynes, MK6 5LD, UK; 2 Royal Gwent Hospital, Department of Obstetrics & Gynaecology, Cardiff Road, Newport, Gwent, NP20 2UB, UK; 3 Homerton University Hospital, Department of Obstetrics & Gynaecology, Homerton Row, Hackney, London, E9 6SR, UK

## Abstract

This case describes a 71 year old, post-menopausal woman who developed vaginal discharge. This complaint ultimately led to the discovery of bowel cancer in conjunction with a large sterile pyometra.

The pyometra was not due to genital malignancy. The most likely conclusion is that the pyometra may have arisen as an inflammatory response to the adjacent bowel pathology. This case report highlights the need for clinicians to consider non-gynaecological cancer as a possible cause for otherwise unexplained pyometra.

## Case presentation

A 71 year old post-menopausal, caucasian woman was admitted under the care of the general surgeons. She was a teetotal pensioner but smoked fifteen cigarettes per day. She had three previous normal vaginal deliveries and her cervical smear history was normal.

She presented with a three day history of feeling generally unwell and left iliac fossa pain. She had no bowel or urinary symptoms. She did however report a generally poor appetite and had lost two stone in weight over the previous six months.

On examination she was febrile and a mass was palpable in the left upper quadrant. A computerized tomography (CT) scan was performed. This showed a 7 cm × 7 cm × 8 cm anomaly on the left side of the abdomen. Diverticuli were also noted in the adjacent bowel.

A colonoscopy was arranged which confirmed the presence of diverticular disease in the sigmoid colon. The clinical impression of the anomaly was that it was a peri-colic abscess and a decision was taken to treat the patient with antibiotics. The patient's symptoms improved and she was discharged home.

A follow up CT scan was requested a few months later but the patient did not attend. She did however attend for surgical out-patient review six months after she first presented. At which time she complained of a greenish vaginal discharge.

She was then referred to the gynaecologists who carried out a pipelle endometrial biopsy and a transvaginal ultrasound scan. This showed a large fluid filled cavity suggesting a pyometra. An urgent hysteroscopy was performed and a large pyometra was confirmed and drained. Endometrial currettings were also obtained. Although the patient had been started on intravenous antibiotics, she subsequently became unwell and febrile.

At this point further imaging was requested. Her CT scan showed a diverticular sigmoid, with a 7 cm collection lying adjacent to the uterus (on the left). It was postulated that there was a fistula between the bowel and the uterus.

The patient's condition continued to deteriorate and she was taken to theatre for an urgent laparotomy. At operation the sigmoid colon mass was found to be adherent to the uterine fundus, with adjacent pus. A subtotal hysterectomy, bilateral salpingo-oophorectomy, left hemi-colectomy and left colostomy were performed by a joint gynaecological – colorectal surgical team. The patient was transferred to the intensive care unit and subsequently made a good post-operative recovery.

Histology showed a moderately differentiated adenocarcinoma of the perforated colon. The uterus showed only a pyometra of uncertain origin and there was absolutely no evidence of a fistula or of a uterine perforation.

## Discussion

Due to the increasing size of the older population, pyometra and its related complications will be encountered by clinicians more frequently [1]. The reported incidence of pyometra is 0.01–0.5% in gynaecological patients but is probably much higher in elderly women.

Pyometra can be caused by a number of gynaecological conditions including puerperal infections, endometrial polyps, leiomyoma's, cervical occlusion after surgery or congenital cervical anomalies. However, the commonest cause of pyometra remains genital tract malignancy and following treatment by radiotherapy [2].

It is rare for a spontaneous perforation of pyometra into the peritoneum, although this does occur [3]. In our patient, the pus in the peritoneal cavity had arisen from perforated bowel, as there was no evidence of any defect in the uterine wall. The pus in the uterine cavity was also sterile, likely to have represented an inflammatory reaction from the adjacent diseased bowel.

## Conclusion

This case highlights the importance of holistic thinking and the learning point is that clinicians should consider that vaginal discharge or indeed pyometra could be a presenting feature of a non-genital malignancy, namely bowel cancer.

## Abbreviations

CT scan: Computerized tomography scan; Cm: Centimetre.

## Competing interests

The authors declare that they have no competing interests.

## Authors' contributions

HS and SW were integrally involved in the patient's management and diagnosis. Both also greatly contributed to writing of the case report. LI performed the literature search and final copy-editing of the paper. All authors read and approved the final manuscript.

## Consent

Written informed consent was obtained from the patient for publication of this case report. A copy of the written consent is available for review by the Editor-in-Chief of this journal.

## References

[B1] Karamercan A, Deniz H, Biri A, Aytac B (2006). Pneumoperitoneum caused by perforation of pyometra without malignancy. Gazi Tip Dergisi.

[B2] Yildizhan B, Uyar E, şiŞmanoğlu A, Güllüoğlu G, Kavak Z (2006). Spontaneous perforation of pyometra. Infect Dis Obstet Gynecol.

[B3] Imachi M, Tanaka S, Ishikawa S, Matsuo K (1993). Spontaneous perforation of pyometra presenting as generalized peritonitis in a patient with cervical cancer. Gynecologic Oncology.

